# Corrected QT Interval (QTc) Diagnostic App for the Oncological Routine: Development Study

**DOI:** 10.2196/48096

**Published:** 2023-09-11

**Authors:** Kristina Klier, Yash J Patel, Timo Schinköthe, Nadia Harbeck, Annette Schmidt

**Affiliations:** 1 Institute of Sport Science University of the Bundeswehr Munich Neubiberg Germany; 2 CANKADO GmbH Ottobrunn Germany; 3 Research Center for Smart Digital Health University of the Bundeswehr Munich Neubiberg Germany; 4 Department of Gynecology and Obstetrics Comprehensive Cancer Center of the Ludwig-Maximilians-University Munich Germany

**Keywords:** telemedicine, mobile health, mHealth, eHealth, tele-cardiology, cardiology, long QT syndrome, prolonged QT interval, electrocardiography, ECG, telehealth, app, application, oncology, cancer, diagnosis, diagnostic, heart, arrhythmia, cardiotoxic, side effects, adverse effects

## Abstract

**Background:**

Numerous antineoplastic drugs such as chemotherapeutics have cardiotoxic side effects and can lead to long QT syndrome (LQTS). When diagnosed and treated in time, the potentially fatal outcomes of LQTS can be prevented. Therefore, regular electrocardiogram (ECG) assessments are critical to ensure patient safety. However, these assessments are associated with patient discomfort and require timely support of the attending oncologist by a cardiologist.

**Objective:**

This study aimed to examine whether this approach can be made more efficient and comfortable by a smartphone app (QTc Tracker), supporting single-lead ECG records on site and transferring to a tele-cardiologist for an immediate diagnosis.

**Methods:**

To evaluate the QTc Tracker, it was implemented in 54 cancer centers in Germany. In total, 266 corrected QT interval (QTc) diagnoses of 122 patients were recorded. Moreover, a questionnaire on routine ECG workflow, turnaround time, and satisfaction (1=best, 6=worst) was answered by the centers before and after the implementation of the QTc Tracker.

**Results:**

Compared to the routine ECG workflow, the QTc Tracker enabled a substantial turnaround time reduction of 98% (mean 2.67, 95% CI 1.72-2.67 h) and even further time efficiency in combination with a cardiologic on-call service (mean 12.10, 95% CI 5.67-18.67 min). Additionally, nurses and patients reported higher satisfaction when using the QTc Tracker. In particular, patients’ satisfaction sharply improved from 2.59 (95% CI 2.41-2.88) for the routine ECG workflow to 1.25 (95% CI 0.99-1.51) for the QTc Tracker workflow.

**Conclusions:**

These results reveal a significant improvement regarding reduced turnaround time and increased user satisfaction. Best patient care might be guaranteed as the exposure of patients with an uncontrolled risk of QTc prolongations can be avoided by using the fast and easy QTc Tracker. In particular, as regular side-effect monitoring, the QTc Tracker app promises more convenience for patients and their physicians. Finally, future studies are needed to empirically test the usability and validity of such mobile ECG assessment methods.

**Trial Registration:**

ClinicalTrials.gov NCT04055493; https://classic.clinicaltrials.gov/ct2/show/NCT04055493

## Introduction

The potentially fatal long QT syndrome (LQTS) is one of the main cardiotoxic side effects of cancer drugs [1], including arsenic trioxide [2-6], selective cyclin-dependent kinase 4 and 6 inhibitors such as ribociclib [7-12], tyrosine kinase inhibitors such as vandetanib [13-18], or histone deacetylase inhibitors such as depsipeptide [19,20]. This syndrome is characterized by a prolongation of the corrected QT interval (QTc), which may induce life-threatening arrhythmias, including torsade de pointes (TdP), and can lead to sudden cardiac death [21]. The outcome of LQTS is not necessarily fatal when diagnosed and treated in time, as the medically induced prolongation of the QTc is reversible [22]. Multiple risk factors may contribute to the development of QTc prolongation. Increasing age as well as female sex, for example, are associated with a higher risk of prolonged QTc [23-27].

The normal average QTc for male individuals is approximately <430 ms, whereas the normal average QTc for female individuals is approximately <450 ms. A borderline QTc can be 431-450 ms for male individuals and 451-470 ms for female individuals, whereas a prolonged QTc is considered >450 ms for male individuals and >470 ms for female individuals [28]. An increased QTc of 10 ms contributes to a 5% to 7% exponential increased risk to develop the life-threatening arrhythmia TdP. Thus, a QTc of 540 ms exposes the patient to a 63% to 97% higher TdP risk than a QTc of 440 ms, but there is no QTc value at which TdP certainly occurs [29-31]. In the case of drug-induced LQTS with a QTc increased to >500 ms or a QTc prolongation of >60 ms above the baseline, treatment discontinuation or alternative therapies should be considered [1].

Regular QTc assessment is associated with additional effort for the attending physician and patient. In the context of oncologic treatments, QTc examination commonly requires the consultation of a cardiologist in addition to the attending oncologist, as many oncologists do not have the ability to record and diagnose electrocardiograms (ECGs) directly on-site. This situation forces the typically older patients with cancer to additionally visit a cardiologist, which in turn exposes them to more stress and endangers susceptible patients. Even if the oncologists can conduct an ECG themselves, this does not guarantee the correct QTc assessment, as only <25% of noncardiologists can correctly classify a QTc as prolonged or normal [32]. Additionally, a 12-lead ECG in general requires the patient to undress and lay down, which is especially uncomfortable for older patients. For example, antiembolism stockings can significantly impede the undressing process for an older patient, and putting the stockings back on after the measurement is often difficult for them [33]. Ultimately, all these mentioned challenges and disadvantages may contribute to the known underuse of ECG monitoring in routine patients in oncology [34]. Therefore, the aim of this study was to examine whether this conventional procedure can be made more efficient and comfortable by a smartphone app (QTc Tracker; version 4.27.30; CANKADO GmbH), supporting single-lead ECG records on site and transferring to a tele-cardiologist for an immediate diagnosis.

## Methods

### Participants and Study Procedure

In total, 54 centers in Germany with 122 patients participated in the study, and 266 QTc diagnoses were recorded. A questionnaire on routine ECG workflow, turnaround time, and satisfaction was answered by the centers before and after the implementation of the QTc Tracker. The turnaround time of the QTc Tracker was accessed from the software itself. All participating patients were diagnosed with early breast cancer and were on ribociclib-based therapy. Since ribociclib can lead to drug-induced LQTS as an adverse drug reaction, patients were monitored by ECG via at least 3 points every 2 weeks at the start of therapy.

### Ethical Considerations

The study was conducted according to the Declaration of Helsinki, and all participants provided informed written consent prior to the measurements. Ethical approval was provided by the West German Study Group (study ID: WSG-AM08). Participation was free and included no further risks. Participants were randomly assigned to numerical codes, so that data could be handled anonymously. The study is part of a registered trial (ClinicalTrials.gov; NCT04055493).

### Questionnaire

The questionnaire used was developed from our own experiences and can be found in Multimedia Appendix 1. It comprised 11 questions about the routine ECG workflow, turnaround time, and satisfaction. Answers were given from nurses’ and patients’ perspectives as free text and rated between 1 (best) and 6 (worst). All centers provided information about their routine ECG workflow and how they routinely receive the QTc diagnosis (paper based or digital).

### KardiaMobile and Kardia App

**Figure 1:**
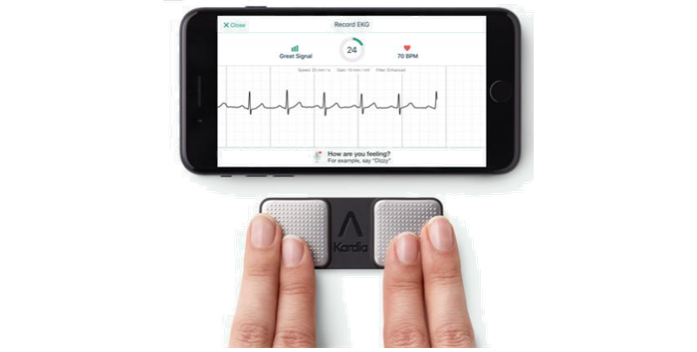
Recording of an electrocardiogram (ECG) with the KardiaMobile Device connected to the Kardia App. The single-lead ECG is recorded by laying the fingers on the electrodes of the KardiaMobile ECG device. A smartphone is connected to the device and records the ECG.

The portable ECG device KardiaMobile (model AC-009; AliveCor Inc) was used to record the single-lead ECGs. The device has US Food and Drug Administration and European Union clearance and Conformité Européenne labeling as a medical device and can be used to calculate QT intervals and monitor drug-mediated QTc prolongation [35-37]. The device comprises 2 electrodes that are used to measure a single-lead ECG between both hands (Figure 1). The ECG recording is transmitted wirelessly to the respective Kardia App (version 5.7.4; AliveCor Inc).

### QTc Tracker

**Figure 2:**
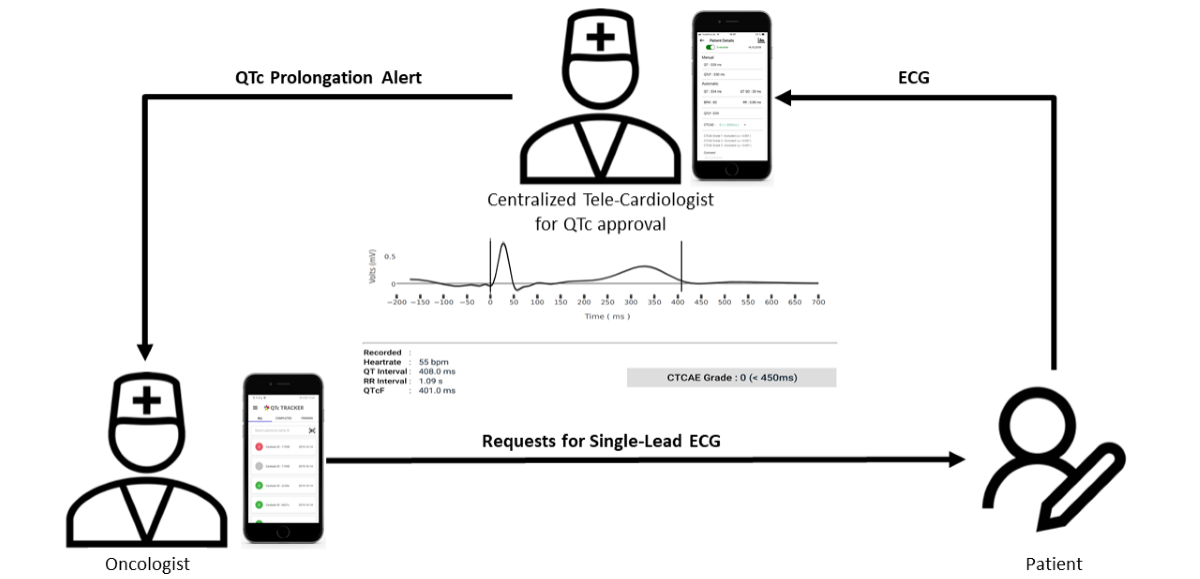
Overview of the general QTc Tracker workflow. Immediate corrected QT intverval diagnosis via smartphone app supporting single-lead ECG record on-site and transfer to tele-cardiologist.

During a routine check-up, the patient records a single-lead ECG on site at the cancer center with the KardiaMobile single-lead ECG device. A PDF file is created with the Kardia App and transferred to the QTc Tracker of the attending oncologist. The assignment of an ECG file to the corresponding patient’s record—either a new or an already existing patient—is guided using a unique patient ID. The QTc Tracker interface for oncologists shows each recorded ECG in an individual row. Next to the patients’ ID is a colored circle, which represents the status of the QTc diagnosis request. If the diagnosis is still pending, the circle is gray. After diagnosis, the circle changes its color according to the Common Terminology Criteria for Adverse Events (CTCAE) grade of the QTc diagnosis. In the case of a normal QTc with a CTCAE grade of 0, the circle is green. If the QTc is pathologic, the color is either orange for a CTCAE grade of 1 or red for a CTCAE grade of 2 or higher (Figure 2).

When the oncologist requests the QTc diagnosis of the ECG file, both the request and the ECG data are transferred to a tele-cardiologist, who then receives a push notification. The QTc Tracker also comprises an interface for the tele-cardiologist (Figure 2). This interface includes an overview of all pending QTc diagnosis requests sorted by the time of receipt. It is structured similar to the interface of the oncologist, with each row representing one request containing the unique patient’s ID and the color-coded circle for the diagnosis status. A gray circle symbolizes a pending ECG diagnosis, whereas a green circle symbolizes the completed diagnosis. The tele-cardiologist is able to open specific requests and view the original ECG file recorded by the Kardia App for revision and diagnosis. Although the QT interval, QT SD, beats per minute, RR interval, QTc using the Fridericia formula (QTcF), and CTCAE grade are calculated automatically, the cardiologist is able to graphically adjust the QTc (Figure 2). The different elements of the ECG are extracted, so the ECG cycles can be identified and further analyzed. For each cardiac cycle, the QT interval is determined according to Martínez and colleagues [38] and corrected for the heart rate with the Fridericia formula (QTcF) as recommended by the European Society of Cardiology [1]. The correction formula according to Fridericia [39] is as follows:


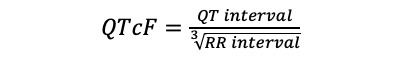


The RR interval is the interval between the R waves of 2 adjacent heart cycles. By determining the QRS onset and T offset (Figure 2), the QTcF is calculated automatically. An overlay plot of all cardiac cycles of an ECG is produced, displaying the mean cardiac cycle and the SD. The mean QTcF is also calculated and displayed.

According to *CTCAE version 5.0* [40,41], QTc prolongations are classified as follows: grade 1 is defined as an average QTc of 450-480 ms; grade 2 is defined as an average QTc of 481-500 ms; grade 3 is defined as an average QTc of >500 ms or a >60 ms change from baseline; and grade 4 is defined as the presence of TdP, polymorphic ventricular tachycardia, or serious arrhythmia [40,41]. After the completion and submission of a diagnosis, the diagnostic report is generated as a PDF file and cannot be changed anymore. Additionally, a comment can be inserted by the tele-cardiologist into the diagnostic report. The details of a diagnostic report are saved in the electronic patient record and transmitted to the oncologist, who subsequently receives a push notification too. The QTc Tracker directly displays the CTCAE grade of a diagnosed ECG in the patient overview. For further details, the oncologist can view the complete diagnostic report. They also have the possibility to download the whole diagnostic report, print it, and add it to the local patient records.

## Results

### General Workflow

The fundamental principle of the telecardiology application QTc Tracker was the on-site ECG measurement at the cancer center and the direct transfer of the ECG data from the smartphone of the oncologist to the tele-cardiologist (Figure 2).

### Routine ECG

The overall results of the questionnaire before the tracker’s implementation are shown in Table 1. Not all centers answered all questions about the turnaround times of their routine ECG assessments. Information about the waiting time for an ECG appointment and the time span between ECG measurement and QTc diagnosis were obtained from 91% (49/54) and 87% (47/54) of the centers, respectively. The total turnaround time was obtained from 81% (45/54) of the centers, wherein both the waiting time for an ECG appointment and the time span between ECG measurement and QTc diagnosis were obtained.

**Table 1:** Overview of the different centers that participated in the study.

Group	Centers (N=54), n (%)	Diagnosis format, n/N (%)		Time to appointment, mean (95% CI)	Time to diagnosis after appointment, mean (95% CI)	Total turnaround time, mean (95% CI)
		Paper based	Digital			
Group 1^a^	21 (39)	21/21 (100)	0/21 (0)	12.45 (2.90-22.00) d	3.79 (0.60-6.97) d	12.77 (2.83-22.70) d
Group 2^b^	9 (17)	6/9 (67)	3/3 (33)	2.64 (0.13-5.14) d	1.14 (0.86-1.42) d	2.16 (0.23-4.08) d
Group 3^c^	24 (44)	21/24 (88)	3/24 (12)	25.08 (15.50-34.65) min	51.98 (18.45-85.51) min	72.52 (36.84-108.20) min
Total	54 (100)	48/54 (89)	6/54 (11)	4.76 (1.12-8.40) d	1.64 (0.34-2.94) d	5.44 (1.43-9.45) d

^a^Group 1: centers without cardiologist.

^b^Group 2: centers with cardiologist who did not receive the corrected QT interval diagnosis on the same day.

^c^Group 3: centers with cardiologist who received the corrected QT interval diagnosis on the same day.

The 54 centers had a mean total turnaround time of 5.44 (95% CI 1.43-9.45) days, which was composed of a mean waiting time for an ECG appointment of 4.76 (95% CI 1.12-8.40) days and a mean time span between ECG measurement and QTc diagnosis of 1.64 (95% CI 0.34-2.94) days. Due to the high variation of the turnaround times, the centers were classified into 3 groups. Group 1 included centers without an in-house cardiologist (21/54, 39% of centers). Group 2 consisted of centers with their own cardiologist who did not receive the QTc diagnosis on the same day (9/54, 17%). Group 3 included centers with their own cardiologist who received the QTc diagnosis on the same day (24/54, 44%).

### QTc Tracker

The second part of the questionnaire about the workflow and turnaround time of using the QTc Tracker was answered by 12 centers. The total turnaround time of the QTc Tracker was retrieved from the system itself and comprised 266 QTc diagnoses, of which 223 (83.8%) were evaluable. Due to the sample size, the QTc Tracker results were not subdivided into groups but evaluated as a whole; therefore, group 4 represents all centers using the QTc Tracker. The mean turnaround time of the QTc Tracker until the receipt of the diagnostic report by the oncologist was 2.67 (95% CI 1.72-2.67) hours. The time reduction from the mean turnaround time of all centers of 5.44 days to 2.67 hours equals to a reduction of 98%.

Further, a cardiologic on-call service was implemented as a trial to perform the QTc diagnosis. This workflow is constituted as group 5. Thereby, the mean turnaround time was further decreased to 12.10 (95% CI 5.67-18.67) minutes. In contrast to the routine ECG workflow, the mean total turnaround time was reduced by over 99%. The combination of the QTc Tracker with a cardiologic on-call service was tested for 28 QTc diagnoses.

### Satisfaction

The majority of the centers (47/54, 87%) completed all questions about their satisfaction. Again, for the questions about the workflow and turnaround time, the second part of the questionnaire about satisfaction with the QTc Tracker workflow was answered by 12 centers.

The overall mean satisfaction grade of the nurses improved from 2.57 (95% CI 2.31-2.84) for the routine ECG workflow to 2.21 (95% CI 1.55-2.86) for the QTc Tracker workflow. The overall mean satisfaction grade of the patients improved from 2.65 (95% CI 2.41-2.88) for the routine ECG workflow to 1.25 (95% CI 0.99-1.51) for the QTc Tracker workflow. Common feedback from the study centers was relief from the patients as it was not necessary to visit a cardiologist in addition to the oncologist. Another positive feedback was the simplicity of the single-lead ECG measurement, especially because the patient does not need to undress for the procedure.

## Discussion

### Principal Findings

Since many medications ranging from antiarrhythmics to oncologic agents may prolong the QTc, it is necessary to search for a more comfortable and time-effective solution to monitor for QTc prolongation. Aside the conventional 12-lead ECG method, smartphone-dependent ECG devices were developed in recent years using a more convenient mode to measure ECGs without the need to undress. The versatility of mobile phones and the general accessibility to the internet enabled the possibility to use newly developed, smartphone-based heart rhythm monitors to assess the QTc directly on site with real-time tele-cardiologic QTc diagnosis, which in turn allows the practitioners to directly react in case of a prolongation.

Previous research confirmed the suitability of using single-lead ECGs recorded by the smartphone-based heart rhythm monitor KardiaMobile to evaluate the QTc in various age and disease groups [35,37,42]. This new technology was shown to be very promising for outpatient QTc monitoring [36]. In general, the market of mobile and smartphone-based ECG devices is constantly expanding, with the perspective that such devices would be implemented into routine care in the next few years [37]. Apart from QTc prolongations as considered in this study, other heart rhythm disturbances can also be diagnosed including atrial fibrillation and atrial flutter [43].

To date, a change in the interaction between attending physicians and cardiologists for QTc diagnosis using single-lead ECGs was not an objective of previous research. The QTc Tracker is the first tele-cardiologic solution for QTc diagnosis using single-lead ECGs recorded by a KardiaMobile device. The field of telemedicine is a constantly evolving science that, according to the World Health Organization, comprises the provision of clinical support by connecting geographically separated users with modern information and communication technologies to improve health outcome [44]. Telemedicine was assessed as an important health care aspect that is under constant progress, with the perspective of it being implemented as a gold-standard technique in the future [45,46]. Currently, the majority of telemedicine systems address the topics of radiology and stroke care [46]. Especially regarding the recent COVID-19 pandemic, telemedicine is becoming more and more relevant [47,48], and therefore, more usable and efficient alternatives are needed.

However, some constraints are limiting our study. The first limitation is the dependency on technical support. In rural regions, internet support in general as well as technical support are not always given as needed for using the QTc Tracker. Another limitation is that we did not assess the accuracy of the measurements in this study but instead aimed for the usability of the assessment method. Therefore, further analysis of the accuracy of the QTc Tracker measurements is necessary. As the tool is promising for regular side-effect monitoring, it is currently integrated into several phase III-IV clinical trials in Germany. In the future, it is planned to use the QTc Tracker not only in combination with a central cardiology service but also to support the centers with their own cardiologists to conduct the diagnosis themselves.

### Conclusions

The QTc Tracker provided a significant improvement for the cancer centers, enabling a highly reduced turnaround time and improved user satisfaction for QTc diagnoses. Finally, future studies should not only establish but also empirically test the usability and validity of such mobile ECG assessment methods.

## Appendix

Multimedia Appendix 1 Questionnaire used in the study.

## Abbreviations

CTCAE: Common Terminology Criteria for Adverse Events
ECG: electrocardiogram
LQTS: long QT syndrome
QTc: corrected QT interval
QTcF: QT interval corrected for heart rate using the Fridericia formula
TdP: torsade de pointes

## Data Availability

The data sets used and/or analyzed during the current study are available from the corresponding author upon reasonable request.
