# Supplementary material for: Corrected QT Interval (QTc) Diagnostic App for the Oncological Routine: Development Study
Source: JMIR Cardio. 2023 Sep 11;7:e48096. doi: 10.2196/48096 (PMC10520775; doi:10.2196/48096)
Supplement: Multimedia Appendix 1 [file cardio_v7i1e48096_app1.docx]

# Questionnaire

| Zentrum |  |
| --- | --- |
| Nurse |  |
| Datum |  |

**EKG Workflow ohne CANKADO:**

1. Haben Sie einen hauseigenen Kardiologen?

| - ja | - nein |
| --- | --- |

1. Falls sie keinen hauseigenen Kardiologen haben, haben Sie einen festen Kardiologen, mit dem Sie zusammenarbeiten?

| - ja | - nein |
| --- | --- |

1. Bitte schätzen Sie den üblichen Zeitraum von der Überweisung zum Kardiologen bis zur Aufnahme des EKG.

|  |
| --- |

1. Bitte schätzen Sie den üblichen Zeitraum zwischen der Aufnahme des EKG und dem Eingang des EKG Befundes bei Ihnen.

| hh |
| --- |

1. In welcher Form erhält der behandelnde Arzt den EKG Befund?

- Fax
- Post
- Direkt digital (Kardiologe ist in der gleichen Praxis/Krankenhaus)
- Patient bringt den Befund zum nächsten Termin mit
- ____________________________________________________________________________

1. Welche Schulnote würden Sie dem **bisherigen** Verfahren geben? Mit den Noten von 1 bis 6.

| - 1 | - 2 | - 3 | - 4 | - 5 | - 6 |
| --- | --- | --- | --- | --- | --- |

1. Welche Schulnote denken Sie würden Patienten geben?

| - 1 | - 2 | - 3 | - 4 | - 5 | - 6 |
| --- | --- | --- | --- | --- | --- |

| Datum |  |
| --- | --- |

**EKG Workflow mit CANKADO und dem KardiaMobile EKG Gerät:**

1. Würden Sie sich wünschen, dass ihr hauseigener Kardiologe die CANKADO QTc Befundung durchführt?

| - ja | - nein |
| --- | --- |

1. Wo schauen Sie sich den QTc Befund an?

- Smartphone
- Computer
- ____________________________________________________________________________

1. Welche Schulnote würden Sie dem **neuen** Verfahren geben? Mit den Noten von 1 bis 6.

| - 1 | - 2 | - 3 | - 4 | - 5 | - 6 |
| --- | --- | --- | --- | --- | --- |

1. Welche Schulnote denken Sie würden Patienten geben?

| - 1 | - 2 | - 3 | - 4 | - 5 | - 6 |
| --- | --- | --- | --- | --- | --- |
